# Assessment of the Glenoid Morphology Based on Demographic Data in the Turkish Population

**DOI:** 10.1155/2020/5736136

**Published:** 2020-02-10

**Authors:** Abdulkadir Sarı, Yaşar Mahsut Dinçel, Burak Günaydın, Mehmet Ümit Çetin, Ömer Özçaglayan, Kerem Bilsel

**Affiliations:** ^1^Namık Kemal University, Faculty of Medicine, Department of Orthopedics and Traumatology, Tekirdağ, Turkey; ^2^Namık Kemal University, School of Medicine, Department of Radiology, Tekirdağ, Turkey; ^3^Bezmialem Vakıf University School of Medicine, Department of Orthopedics and Traumatology, Istanbul, Turkey

## Abstract

**Purpose:**

In this study, our aim was to evaluate the glenoid version, height, and width measurements based on gender, side, age, height, and hand dominance in the Turkish population using computed tomography (CT) images.

**Methods:**

In our study, CT images of 140 patients (62 females and 78 males; mean age: 39.6 years) who had no shoulder complaints were evaluated retrospectively. Glenoid version (GV), AP diameter (width), and SI diameter (height) on both shoulders were measured on the CT images. Correlations between patient gender, side, age, height, and hand dominance and the GV and size were evaluated.

**Results:**

The right shoulder had a mean GV of −0.93 ± 7.80 degrees and the left shoulder had a GV of −0.88 ± 6.63 degrees (*p* > 0.05). The mean AP diameter of the glenoid was 26.57 ± 3.02 mm in the right shoulder and 26.33 ± 3.01 mm in the left shoulder (*p* > 0.05). The mean AP diameter of the glenoid was 26.57 ± 3.02 mm in the right shoulder and 26.33 ± 3.01 mm in the left shoulder (*p* > 0.05). The mean AP diameter of the glenoid was 26.57 ± 3.02 mm in the right shoulder and 26.33 ± 3.01 mm in the left shoulder (*p* > 0.05). The mean AP diameter of the glenoid was 26.57 ± 3.02 mm in the right shoulder and 26.33 ± 3.01 mm in the left shoulder (*p* > 0.05). The mean AP diameter of the glenoid was 26.57 ± 3.02 mm in the right shoulder and 26.33 ± 3.01 mm in the left shoulder (*p* > 0.05). The mean AP diameter of the glenoid was 26.57 ± 3.02 mm in the right shoulder and 26.33 ± 3.01 mm in the left shoulder (*p* > 0.05). The mean AP diameter of the glenoid was 26.57 ± 3.02 mm in the right shoulder and 26.33 ± 3.01 mm in the left shoulder (*p* > 0.05). The mean AP diameter of the glenoid was 26.57 ± 3.02 mm in the right shoulder and 26.33 ± 3.01 mm in the left shoulder (

**Conclusion:**

Hand dominance had an effect on the glenoid version, while patient gender, age, and height had an effect on the glenoid size. The glenoid width in the Turkish population was similar to that of the European and American populations, and the glenoid height was similar to that of the Asian population. Our GV values were similar to those of the Asian population and more anteverted compared to the Western population. We believe that our findings will be useful in preoperative planning and in the production of implants for our population.

## 1. Introduction

Recognition of the glenoid anatomy is extremely important in surgical restoration of the natural anatomy, as well as for our understanding of instability and degenerative processes [1–5].

Contrary to popular belief, many studies on morphology have shown that all individuals had great variations in the glenoid rather than having a neutral or retroverted anatomy [6–8]. Gender, hand dominance, height in patients, and ethnic differences were observed to have an effect on the glenoid size and version [1, 9]. It is therefore important to have the isolated data of the Turkish population.

In their study of more than 300 glenoids, Churchill et al. [10] found no differences between the glenoid size in Afro-Americans and whites, but the versions were statistically different. However, the versions of men and women of the same race were not different.

In Piponov et al.'s study [1], gender and ethnicity were found to have an effect on the glenoid size and version. Hispanics had on average 6.4 degrees more anteverted glenoids compared to Afro-Americans, and men had more retroverted and bigger glenoids compared to women.

In our study, we aimed at evaluating the glenoid version (GV), height, and width measurements based on gender, side, age, height, and hand dominance in the Turkish population using computed tomography (CT) images.

## 2. Materials and Methods

Our study was approved by the ethics committee of our faculty. Both shoulders of the subjects were evaluated retrospectively on thoracic CT images in the PAC system of our hospital. The patients were interviewed on the phone, and those between the ages of 18 and 60 years, who had no degenerative, traumatic, or congenital shoulder problems, and who did not undergo a surgery of the shoulder region were included in the study. None of the cases had shoulder complaints. When determining the dominant hand, the hand primarily used in daily activities was taken into consideration. In order to include more individuals with left-hand dominance, a total of 1500 subjects were scanned. Among them, 140 cases who complied with the above criteria were included in our study.

The measurements were made collectively by an orthopedist and a musculoskeletal radiologist using axial and coronal sections on CT sections that included both shoulders. The thorax CT scans were performed using a Toshiba Aquilion™ PRIME 80 scanner (Toshiba Medical Systems Corp., Tokyo, Japan). The thorax CT parameters were as follows: kV: 120; maS: 80; collimation: 1.25 × 1.25 mm; pitch: 1; FOV: 20 × 20 cm; matrix: 512 × 512; and slice thickness: 1 mm. Raw data were processed by an experienced radiologist on the Sectra PACS system (Sectra AB, Linköping, Sweden), and multiplanar reconstruction (MPR) images were obtained.

**Figure 1:**
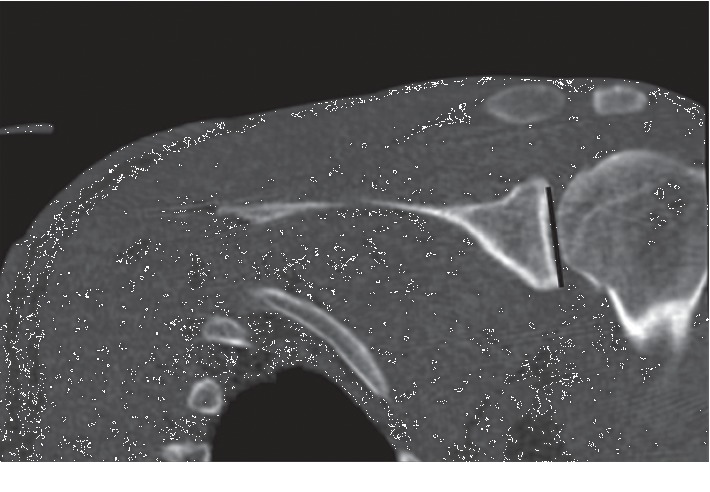
Measurement of the SI diameter (height) of the glenoid on the coronal CT image.

**Figure 2:**
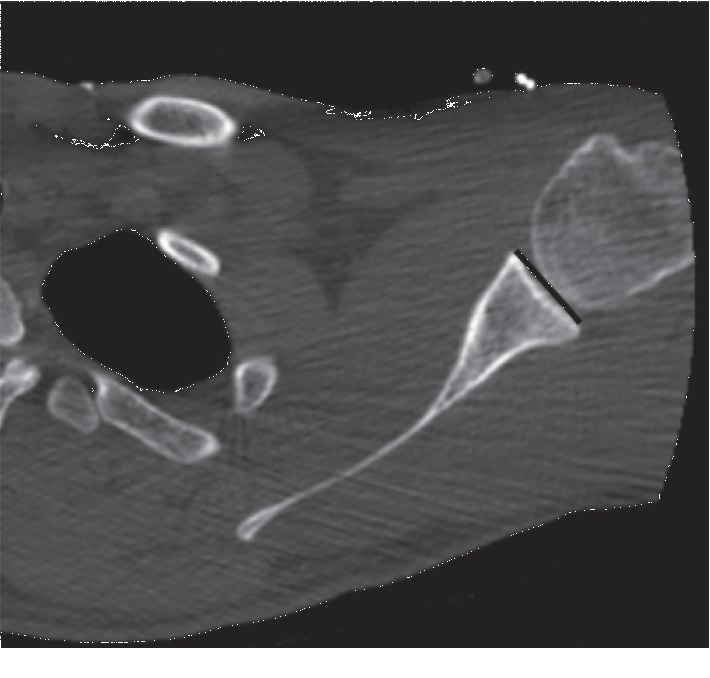
Measurement of the AP diameter (width) of the glenoid on the axial CT image.

**Figure 3:**
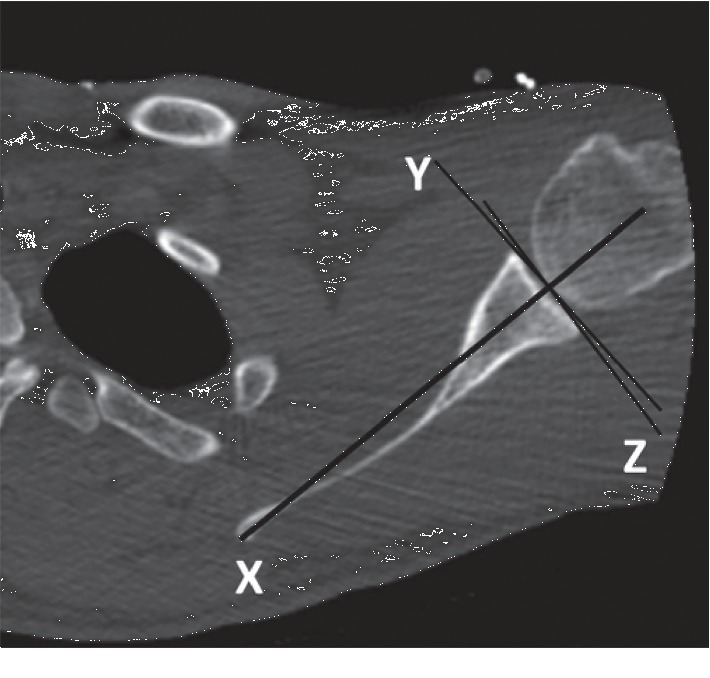
Measurement of the glenoid version on the axial CT image. *X*: scapular axis (Friedman line); *Y*: line of neutral version; *Z*: glenoid axis. The version angle is the angle formed between the *Y* and *Z* lines.

The GV, AP diameter (width), and SI diameter (height) of the glenoids in both shoulders were compared according to the method described by Friedman et al. [11]. The highest values were considered as the glenoid height and width (Figures 1 and 2). In measurement of the version, the neutral rotation line was determined according to the scapular axis first. The angle between the glenoid axis and the neutral rotation line was accepted as the degree of the version (Figure 3). Accordingly, the anteversion values were recorded as positive and the retroversion values as negative.

The side, age, gender, height, and hand dominance were recorded to be used in the comparisons.

Mean, standard deviation, median, minimum, maximum, frequency, and percentage values were used in the descriptive statistics of the data. The distribution of the variables was evaluated using the Kolmogorov–Smirnov test. The Mann–Whitney *U* test was used to analyze the independent quantitative data and the Wilcoxon test was utilized in the analysis of the dependent quantitative data. Spearman's correlation analysis was employed in correlation analysis. The SPSS v.22.0 (IBM Corp., Armonk, NY, USA) software was used in the analyses. The significance level was set at *p* < 0.05.

## 3. Results

A total of 140 patients, 62 females (44.3%) and 78 males (55.7%), were included in our study. The patients had a mean age of 39.6 ± 11.8 (range: 18 to 60 years) and a mean height of 170.5 ± 8.2 (range: 152 to 193 cm). The right hand was dominant in 64.3% (*n* = 90) while the left hand was in 35.7% (*n* = 50) of the cases (Table 1). The right shoulder had a mean GV of −0.93 ± 7.80 degrees and the left shoulder had a GV of −0.88 ± 6.63 degrees (*p* > 0.05). The mean AP diameter of the glenoid was 26.57 ± 3.02 mm in the right shoulder and 26.33 ± 3.01 mm in the left shoulder (*p* > 0.05). The mean SI diameter of the glenoid was 31.8 ± 3.6 mm in the right and 31.7 ± 3.3 mm in the left shoulder (*p* > 0.05) (Table 2).

The right glenoid was significantly more retroverted than the left glenoid in the right-hand-dominant group and the left glenoid was significantly more retroverted than the right glenoid in the left-hand-dominant group (*p* < 0.05). No correlation was detected between hand dominance and the AP and SI diameters in both shoulders (*p* > 0.05) (Table 3).

There was no significant correlation between age and the GV values in both shoulders (*p* > 0.05). A positive correlation was observed between the AP and SI diameters of the glenoid in both shoulders (*p* < 0.05). There was no correlation between the heights of the patients and the GV values in both shoulders (*p* > 0.05). However, both the AP and SI diameters in both shoulders correlated positively with the heights of the patients (*p* < 0.05) (Table 4).

When males and females were evaluated in two separate groups, the GV, AP, and SI values did not exhibit a statistically significant difference between the two shoulders in both genders (*p* > 0.05) (Table 5).

The GV values in the right and left shoulders did not differ significantly between the male and female groups (*p* > 0.05). In evaluating the effect of gender on the AP and SI diameters, we found out that the male group had significantly larger AP and SI diameters of the glenoid (*p* < 0.05) (Table 6).

There was no significant relationship between the ages and heights of the patients and the GV values in females (*p* > 0.05). However, there was a positive correlation between the ages and heights of the patients and the AP and SI diameters (*p* < 0.05). No relationship was detected between the ages and heights of the patients and the GV values in males (*p* > 0.05). Yet, a positive correlation between the ages and heights of the patients and the AP and SI diameters was observed (*p* < 0.05).

## 4. Discussion

Understanding of the glenoid anatomy is still ongoing in our day [6, 7, 12, 13]. Studies have shown that the GV and size vary among different ethnic groups [1, 5, 10, 14, 15]. To the best of our knowledge, no study has been performed to reveal the glenoid morphology in healthy individuals in the Turkish population.

Our study demonstrated that the average GV value was −0.93 in the right shoulder and −0.88 degrees in the left shoulder. Our measurements of the GV were close to −1 degrees, as indicated in previous studies [1, 3, 10, 16, 17]. Matsumura et al. [16] reported a positive correlation between retroversion and the male gender and hand dominance (*p* < 0.001) and found a difference of 1 degree between the shoulders. However, 11 left-handed individuals were included in their study. Piponov et al. [1] found that gender had an impact on the version and that men had more retroverted glenoids with an average of 3 degrees. However, the authors did not take hand dominance into account in their study. We scanned a huge group of patients from our society due to the low possibility of finding left-hand-dominant individuals, and we were able to include only 50 left-handed individuals in our study. Our cases had similar distributions in terms of gender, age, and hand dominance. In our study, a retroversion of 1.07 degrees and 1.04 degrees was detected in favor of the dominant side in the right-hand-dominant and left-hand-dominant individuals, respectively (*p* < 0.05). The gender parameter did not have an effect on the version; the mean right GV values showed 1 degree and the left GV values 0.8 degrees of difference between males and females (*p* > 0.05). Similarly, the literature holds several studies that did not establish a relationship between gender and version, in accordance with our results [11, 18–20]. In another study of Matsumura et al., the authors did not detect a relationship between GV and gender, and they observed a mean version of −1 ± 4 and 0 ± 4 degrees in males and females, respectively (*p*=0.059) [12]. However, only five left-handed individuals were included in their study. Matsuki et al. [21] formed similar groups of patients in terms of gender and side but did not evaluate hand dominance. The difference of 1 degree detected between the GV values of the genders (−2.2 ± 6.4 degrees in males vs. −3.2 ± 3.9 degrees in females) was considered statistically insignificant (*p* > 0.05). We could not detect any relationship between GV and the ages and heights of our cases.

In our study, the AP and SI values did not differ between the left and right glenoids of the cases. However, the genders, heights, and ages of the patients were found to have an effect on the AP and SI values. The mean AP and SI diameters were 28 mm and 34 mm in males and 24 mm and 30 mm in females, respectively (*p* < 0.05). The mean AP and SI values in both shoulders were 4 mm larger in males. Matsumura et al. similarly reported a 4 mm difference between the AP and SI values in male and female subjects (*p* < 0.001) [12].

Shi et al. studied a Chinese population and found a mean AP diameter of 28.5 mm and SI diameter of 37 mm in males and a mean AP diameter of 25 mm and SI diameter of 33 mm in females [14]. In a study conducted on an American population, Merrill et al. measured the AP diameter as 28.56 mm and the SI diameter as 37.01 mm in males, and the AP diameter as 24 mm and the SI diameter as 34 mm in females [20]. The similarity of the results between the studies of Shi et al. and Merrill et al. is remarkable. It is known that the glenoid size in the Asian population is smaller than that in the American and European counterparts [12, 21, 22]. Here, the age factor, the second determinant as we also noticed in our study, steps forward. In Shi et al.'s study, the male participants had an average age of 55 while the females had an average age of 60 [14]. In Merrill et al.'s study, the age of all participants ranged between 30 and 40 years [20]. The average age of the participants in our study was 40, and we detected a direct correlation between age and the glenoid size. In the literature, as pointed out by Matsuki et al. and Bockmann et al., we excluded the osteopathic cases from our study to avoid the inclusion of cases with arthrosis [21, 23]. Still, the correlation detected may be due to overlooked small osteophytes or the subchondral changes in the early period.

Piponov et al. reported a positive correlation between the heights of the patients and the AP and SI values [1]. Similarly, Matsumura et al. found a correlation between the heights of the patients and the glenoid size [12]. In our case, males were 11 cm longer than females. We detected a correlation between the heights of our patients and the AP and SI diameters, in accordance with the literature (*p* < 0.05). We did not observe any relationship between hand dominance and the AP and SI values; the groups showed similar results in terms of size (*p* > 0.05).

The glenoid exhibits different morphological characteristics among ethnic communities [1, 10]. In a recent study by Mizuno et al. [22], the glenoid morphologies of the Japanese and French individuals with similar distributions of gender and age were compared and the AP and SI diameters in the Japanese individuals were found to be 2 mm smaller. In addition, the Japanese had a mean GV of −2.3 degrees and the French −6.0 degrees (*p* > 0.05). The difference in the AP and SI values here may be due to the fact that the French individuals were 4 cm taller than their counterparts, as suggested by the authors. However, this does not explain the ethnic difference between the GV values. In another study, Aygün et al. [5] examined the relationship between shoulder instability and the glenoid morphology in the Turkish population. However, this study was conducted on a smaller population and is far from describing the morphology in healthy individuals. In addition, the distribution of the variables such as patient gender, hand dominance, side, age, and height, which may have a possible effect on the glenoid morphology, and their correlation with the measurement results were excluded. The glenoid was examined in terms of version, but parameters such as glenoid width and height were not investigated. If we would like to compare the values from the Turkish population with the other population values from the literature, we can see that our AP measurements were 3 mm larger, although our SI results were similar in both males and females to those of the Japanese population. Our GV results were also similar to those of the Japanese population; it ranged between neutral and 1 degree of retroversion [12]. In our comparison to the American population, our AP values were similar in both genders, while our SI values were 4 mm smaller [20]. The GV in the Anglo-Saxon literature ranges between −2 and −9 degrees [8, 10, 11]. Our version values were more anteverted compared to these values.

The correct positioning of the glenoid component in prosthetic surgery is extremely important for the prevention of early loosening of the component [6, 24–27]. In addition, the selected implant sizes and designs should be compatible with the morphological characteristics of different populations [21, 28]. European and American-based prosthetic products are often inadequate in meeting the morphological characteristics of the non-Western societies [12]. Therefore, it is very important to reveal the social differences that govern production.

Glenoid morphology still remains a mystery even though the studies to date give a lot of information. As much as ethnic differences, several details such as the imaging method, the measurement technique, choosing the cases for comparison from identical groups, and including all indicators that may have an impact on the results in the study affect the results.

Our study had some limitations. First, we performed our measurements on CT images, which are better known to demonstrate the bone structure [29]. The Friedman method we used is known to be correlated with other current measurement methods [6, 30]. The Friedman and the more recent vault methods present similar results in measuring the version at the middle and lower glenoid; however, the vault method is less reliable and displays more variability [13]. Our second limitation was the age range of 18–60 years. A study with younger patients may reveal the possible effect of age on the glenoid size more clearly. Third, we only examined the glenoid morphology; the morphology of the humeral component of the glenohumeral joint and its orientation with the glenoid could be evaluated. The lack of assessing the intraobserver and interobserver reliability and the lack of recording the indication for CT were other limitations. Finally, we tried to reveal the morphology of the joint only by evaluating the bone structure. The inclusion of bone marrow cartilage in the morphological examination could give us more information.

Our results showed that the differences between the versions in each shoulder were mostly around 1 degree and below, an insignificant value which may be ignored. The difference between the AP and SI values of both glenoids was less than 1 mm. This formal and dimensional similarity between both glenoids indicates that the glenoid values of the contralateral shoulder can be used as a reference in arthroplasty or osteosynthesis. We believe that our study results will also contribute to the determination of the implant sizes compatible with Turkish glenoids and to the prosthetic design process.

In our study, CT images of 140 healthy subjects were analyzed. The largest number of left-hand cases in the literature was included in the study in order to reveal the effect of the side and hand dominance. We found out that hand dominance had an effect on version, and patient gender and height had an effect on the dimensions. We observed that the glenoid width in the Turkish population was similar to that from the European and American literature and that the glenoid height was similar to that from the Asian literature. Our GV values were less retroverted compared to the Western values and similar to the Asian values. We believe that our findings will be useful in preoperative planning and in the production of implants for our population.

## Figures and Tables

**Table 1:** Descriptive statistics of the patients.

		Minimum–maximum	Median	Mean ± SD/*n*, (%)	
Age		18–61	39	39.6 ± 11.8	
Gender	Female			62	44.3%
	Male			78	55.7%
Boy		152–193	170	170.5 ± 8.2	
Hand dominance	Right			90	64.3%
	Left			50	35.7%

**Table 2:** The version, AP, and SI values in both shoulders in both groups.

	Minimum–maximum	Median	Mean ± SD	*p*
*Glenoid version*				
Right side	−18.6–19.4	−1.2	−0.93 ± 7.80	0.150^*∗*^
Left side	−18.6–17.0	−0.6	−0.88 ± 6.63	

*Shoulder AP*				
Right side	20.0–36.0	26.0	26.57 ± 3.02	0.063^*∗*^
Left side	21.0–34.0	26.0	26.33 ± 3.01	

*Shoulder SI*				
Right side	23.0–39.0	31.8	31.8 ± 3.6	0.651^*∗*^
Left side	22.5–39.9	32.0	31.7 ± 3.3	

AP: anteroposterior; SI: superoinferior. ^*∗*^Wilcoxon test. Negative values indicate retroversion.

**Table 3:** Comparison of the version, AP, and SI values based on hand dominance.

	Right-hand-dominant		Left-hand-dominant	
	Mean ± SD	Median	Mean ± SD	Median
*Glenoid version*				
Right side	−1.59 ± 7.18	−0.75	−0.25 ± 8.75	−1.35
Left side	−0.52 ± 6.01	0.00	−1.29 ± 7.02	−1.82
Difference between the right-left sides *p*	0.001^*∗*^		0.001^*∗*^	

*Shoulder AP*				
Right side	26.9 ± 3.2	26.7	26.0 ± 2.6	26.0
Left side	26.6 ± 2.8	27.0	25.8 ± 3.0	26.0
Difference between the right-left sides *p*	0.052^*∗*^		0.618^*∗*^	

*Shoulder SI*				
Right side	31.2 ± 3.8	31.1	33.0 ± 3.1	33.0
Left side	31.1 ± 3.7	30.8	33.0 ± 2.9	33.0
Difference between the right-left sides *p*	0.570^*∗*^		0.953^*∗*^	

AP: anteroposterior, SI: superoinferior. ^*∗*^Wilcoxon test. Significant *p* values are written in bold. Negative values indicate retroversion.

**Table 4:** Correlations of patient age and height with the measurement results calculated with Spearman's correlation.

	Age		Height (cm)	
	*r*	*p*	*r*	*p*
*Glenoid version*				
Right side	0.009	0.920	−0.024	0.780
Left side	0.033	0.647	−0.133	0.116

*Shoulder AP*				
Right side	0.189	**0.026**	0.464	**0.001**
Left side	0.204	**0.016**	0.503	**0.001**

*Shoulder SI*				
Right side	0.894	**0.043**	0.468	**0.001**
Left side	0.941	**0.032**	0.531	**0.001**

AP: anteroposterior, SI: superoinferior. Significant *p* values are written in bold.

**Table 5:** Measurement results in males and females.

	Minimum–maximum	Median	Mean ± SD	*p*
Females				

*Glenoid version*				
Right side	−18.6–19.0	−1.5	−1.68 ± 7.70	0.134^*∗*^
Left side	−18.6–12.0	0.0	−1.13 ± 7.16	

*Shoulder AP*				
Right side	20.0–29.5	24.2	24.51 ± 2.15	0.152^*∗*^
Left side	21.0–29.0	24.0	24.27 ± 2.00	

*Shoulder SI*				
Right side	23.0–34.5	30.0	29.4 ± 2.7	0.783^*∗*^
Left side	22.5–35.0	29.0	29.4 ± 2.7	
Males				

*Glenoid version*				
Right side	−18.0–19.4	−1.1	−0.68 ± 6.22	0.552^*∗*^
Left side	−14.0–17.0	−1.0	−0.34 ± 7.87	

*Shoulder AP*				
Right side	22.0–36.0	28.0	28.21 ± 2.59	0.051^*∗*^
Left side	21.0–34.0	28.0	27.97 ± 2.42	

*Shoulder SI*				
Right side	25.0–39.0	34.0	33.8 ± 3.1	0.429^*∗*^
Left side	24.0–39.9	34.0	33.7 ± 3.0	

AP: anteroposterior, SI: superoinferior.^*∗*^Wilcoxon test. Negative values indicate retroversion.

**Table 6:** Comparison of the measurement results in both shoulders based on gender.

		Female			Male			*p*
		Mean ± SD/*n*, (%)		Median	Mean ± SD/*n*, (%)		Median	
Age		40.0 ± 11.2		39.0	39.4 ± 12.4		39.0	0.704^*∗*^
Height		164.4 ± 5.8		164.0	175.3 ± 6.4		176.0	**0.001 ** ^*∗*^
Hand dominance	Right	44.0	0.7		46.0	0.7		0.141^†^
	Left	18.0	0.3		32.0	0.5		

*Glenoid version*								
Right side		−1.7 ± 7.7		−1.5	−0.7 ± 6.2		−1.1	0.464^*∗*^
Left side		−1.1 ± 7.2		0.0	−0.3 ± 7.9		−1.0	0.953^*∗*^
Difference between the right-left sides *p*		0.134^‡^			0.552^‡^			

*Shoulder AP*								
Right side		24.5 ± 2.1		24.2	28.2 ± 2.6		28.0	**0.001 ** ^*∗*^
Left side		24.3 ± 2.0		24.0	28.0 ± 2.4		28.0	**0.001 ** ^*∗*^
Difference between the right-left sides *p*		0.152^‡^			0.051^‡^			

*Shoulder SI*								
Right side		29.4 ± 2.7		30.0	33.8 ± 3.1		34.0	**0.001 ** ^*∗*^
Left side		29.4 ± 2.7		29.0	33.7 ± 3.0		34.0	**0.001 ** ^*∗*^
Difference between the right-left sides *p*		0.783^‡^			0.429^‡^			

AP: anteroposterior, SI: superoinferior. ^*∗*^Mann–Whitney *U* test. ^†^Chi-square test. ^‡^Wilcoxon test. Significant *p* values are written in bold. Negative values indicate retroversion.

## Data Availability

The dataset used to support the findings of this study is available from the corresponding author upon request.

## References

[1] Piponov H. I., Savin D., Shah N. (2016). Glenoid version and size: does gender, ethnicity, or body size play a role?. *International Orthopaedics*.

[2] Wirth M. A., Seltzer D. G., Rockwood C. A. (1994). Recurrent posterior glenohumeral dislocation associated with increased retroversion of the glenoid. a case report. *Clinical Orthopaedics and Related Research*.

[3] Hohmann E., Tetsworth K. (2015). Glenoid version and inclination are risk factors for anterior shoulder dislocation. *Journal of Shoulder and Elbow Surgery*.

[4] Waters P. M., Smith G. R., Jaramillo D. (1998). Glenohumeral deformity secondary to brachial plexus birth palsy. *The Journal of Bone & Joint Surgery*.

[5] Aygün Ü., Çalik Y., Işik C., Şahin H., Şahin R., Aygün D. Ö. (2016). The importance of glenoid version in patients with anterior dislocation of the shoulder. *Journal of Shoulder and Elbow Surgery*.

[6] Poon P. C., Ting F. S. H. (2012). A 2-dimensional glenoid vault method for measuring glenoid version on computed tomography. *Journal of Shoulder and Elbow Surgery*.

[7] Boileau P., Cheval D., Gauci M.-O., Holzer N., Chaoui J., Walch G. (2018). Automated three-dimensional measurement of glenoid version and inclination in arthritic shoulders. *The Journal of Bone and Joint Surgery*.

[8] Nyffeler R. W., Jost B., Pfirrmann C. W. A., Gerber C. (2003). Measurement of glenoid version: conventional radiographs versus computed tomography scans. *Journal of Shoulder and Elbow Surgery*.

[9] Iannotti J. P., Weiner S., Rodriguez E. (2015). Three-dimensional imaging and templating improve glenoid implant positioning. *The Journal of Bone and Joint Surgery-American Volume*.

[10] Churchill R. S., Brems J. J., Kotschi H. (2001). Glenoid size, inclination, and version: an anatomic study. *Journal of Shoulder and Elbow Surgery*.

[11] Friedman R. J., Hawthorne K. B., Genez B. M. (1992). The use of computerized tomography in the measurement of glenoid version. *The Journal of Bone & Joint Surgery*.

[12] Matsumura N., Oki S., Ogawa K. (2016). Three-dimensional anthropometric analysis of the glenohumeral joint in a normal Japanese population. *Journal of Shoulder and Elbow Surgery*.

[13] Cunningham G., Freebody J., Smith M. M. (2018). Comparative analysis of 2 glenoid version measurement methods in variable axial slices on 3-dimensionally reconstructed computed tomography scans. *Journal of Shoulder and Elbow Surgery*.

[14] Shi L., Griffith J. F., Huang J., Wang D. (2013). Excellent side-to-side symmetry in glenoid size and shape. *Skeletal Radiology*.

[15] Baten J., Blum M., van Zanden J. (2014). Human height since 1820. *How Was Life? Global Well-Being since 1820*.

[16] Matsumura N., Ogawa K., Kobayashi S. (2014). Morphologic features of humeral head and glenoid version in the normal glenohumeral joint. *Journal of Shoulder and Elbow Surgery*.

[17] Kwon Y. W., Powell K. A., Yum J. K., Brems J. J., Iannotti J. P. (2005). Use of three-dimensional computed tomography for the analysis of the glenoid anatomy. *Journal of Shoulder and Elbow Surgery*.

[18] Tackett J. J., Ablove R. H. (2011). Magnetic resonance imaging study of glenohumeral relationships between genders. *Journal of Shoulder and Elbow Surgery*.

[19] von Schroeder H. P., Kuiper S. D., Botte M. J. (2001). Osseous anatomy of the scapula. *Clinical Orthopaedics and Related Research*.

[20] Merrill A., Guzman K., Miller S. L. (2009). Gender differences in glenoid anatomy: an anatomic study. *Surgical and Radiologic Anatomy*.

[21] Matsuki K., Sugaya H., Hoshika S (2018). Three-dimensional measurement of glenoid dimensions and orientations. *Journal of Orthopaedic Science*.

[22] Mizuno N., Nonaka S., Ozaki R., Yoshida M., Yoneda M., Walch G. (2017). Three-dimensional assessment of the normal Japanese glenoid and comparison with the normal French glenoid. *Orthopaedics & Traumatology: Surgery & Research*.

[23] Bockmann B., Soschynski S., Lechler P. (2016). Age-dependent variation of glenohumeral anatomy: a radiological study. *International Orthopaedics*.

[24] Favre P., Sussmann P. S., Gerber C. (2010). The effect of component positioning on intrinsic stability of the reverse shoulder arthroplasty. *Journal of Shoulder and Elbow Surgery*.

[25] Clavert P., Millett P. J., Warner J. J. P. (2007). Glenoid resurfacing: what are the limits to asymmetric reaming for posterior erosion?. *Journal of Shoulder and Elbow Surgery*.

[26] Farron A., Terrier A., Büchler P. (2006). Risks of loosening of a prosthetic glenoid implanted in retroversion. *Journal of Shoulder and Elbow Surgery*.

[27] Büchler P., Ramaniraka N. A., Rakotomanana L. R., Iannotti J. P., Farron A. (2002). A finite element model of the shoulder: application to the comparison of normal and osteoarthritic joints. *Clin Biomech (Bristol, Avon)*.

[28] Athwal G. S., Faber K. J. (2016). Outcomes of reverse shoulder arthroplasty using a mini 25-mm glenoid baseplate. *International Orthopaedics*.

[29] Lenart B. A., Freedman R., van Thiel G. S. (2014). Magnetic resonance imaging evaluation of normal glenoid length and width: an anatomic study. *Arthroscopy: The Journal of Arthroscopic & Related Surgery*.

[30] Andrin J., Macaron C., Pottecher P. (2016). Determination of a new computed tomography method for measuring the glenoid version and comparing with a reference method. Radio-anatomical and retrospective study. *International Orthopaedics*.

